# Latent Profile Analysis of Physical Environmental Characteristics of Older Adults and Its Effect on Intrinsic Capacity

**DOI:** 10.1002/alz70860_100080

**Published:** 2025-12-23

**Authors:** Young Myoung Lim, WonSang Min, Ah‐Ram Kim, Ji‐Hyuk Park

**Affiliations:** ^1^ New Normal Lifestyle Research Institute / Yonsei University, Wonju, Gangwondo, Korea, Republic of (South); ^2^ New Normal Lifestyle Research Institute / Yonsei University, Wonju‐si, Gangwondo, Rep of Korea, Gangwondo, Korea, Republic of (South); ^3^ Yonsei University, Wonju, Gangwon‐do, Korea, Republic of (South)

## Abstract

**Background:**

The aging population is a pressing global issue that necessitates improving older adults' health and quality of life. The physical environment, encompassing residential and community‐level factors, significantly influences older adults' independence and intrinsic capacity (IC). While prior studies address physical environment characteristics, limited research integrates these with multidimensional IC determinants, requiring further investigation.

**Method:**

A cross‐sectional study analyzed data from the 2023 Korean Older Adults Survey (*n* = 7,820). Using latent profile analysis (LPA), we categorized older adults into three latent physical environment groups based on residential accessibility, community mobility, and community friendliness. Multinomial logistic regression and logistic regression evaluated demographic predictors and their associations with IC, measured by six core domains (e.g., sensory and cognitive function) based on WHO's ICOPE framework.

**Result:**

He study identified three latent profiles of physical environment characteristics among older adults. The Restricted Environment group (18.7%) showed the lowest scores across residential accessibility, community mobility, and friendliness, reflecting significant limitations. The Regional Environment group (61.5%) had high community mobility and friendliness but the lowest residential accessibility. The Unbalanced Environment group (19.8%) exhibited the highest residential accessibility with moderate community mobility and friendliness, indicating environmental imbalances. Intrinsic capacity (IC) findings from logistic regression analysis revealed that individuals in the “Regional Environment” group had 29% lower odds (OR=0.71, 95% CI=0.61–0.84) of maintaining high IC compared to those in the “Unbalanced Environment” group. Furthermore, older age was associated with decreased odds of maintaining high IC (e.g., OR=0.21, 95% CI=0.16–0.27 for minimal education), while higher educational attainment significantly increased the likelihood of preserving IC (e.g., OR=1.51, 95% CI=1.40–1.65 for high education). These findings underscore the critical role of physical environments and demographic factors in determining IC.

**Conclusion:**

Physical environment significantly affects older adults' IC, with restricted environments posing greater risks. Tailored interventions, such as improving community mobility and reducing isolation, are critical for enhancing quality of life and health outcomes. These findings provide a foundation for targeted policy and environmental design to support aging populations.

